# Supplementary material for: Probiotics Enhance Bone Growth and Rescue BMP Inhibition: New Transgenic Zebrafish Lines to Study Bone Health
Source: Int J Mol Sci. 2022 Apr 26;23(9):4748. doi: 10.3390/ijms23094748 (PMC9102566; doi:10.3390/ijms23094748)

Probiotics enhance bone growth and rescue BMP inhibition: new transgenic zebrafish lines to study bone health

Jerry Maria Sojan[1+], Ratish Raman[2+], Marc Muller[2*], Oliana Carnevali[1], Jörg Renn[2]

Supplementary Figure S1: Representative images of **(a)** lateral and **(b)** ventral views of zebrafish larvae head showing how the pixel intensity was measured in both views. Red indicate the area measured and yellow denote the eye area substracted. Bony structures are in white against grey background.

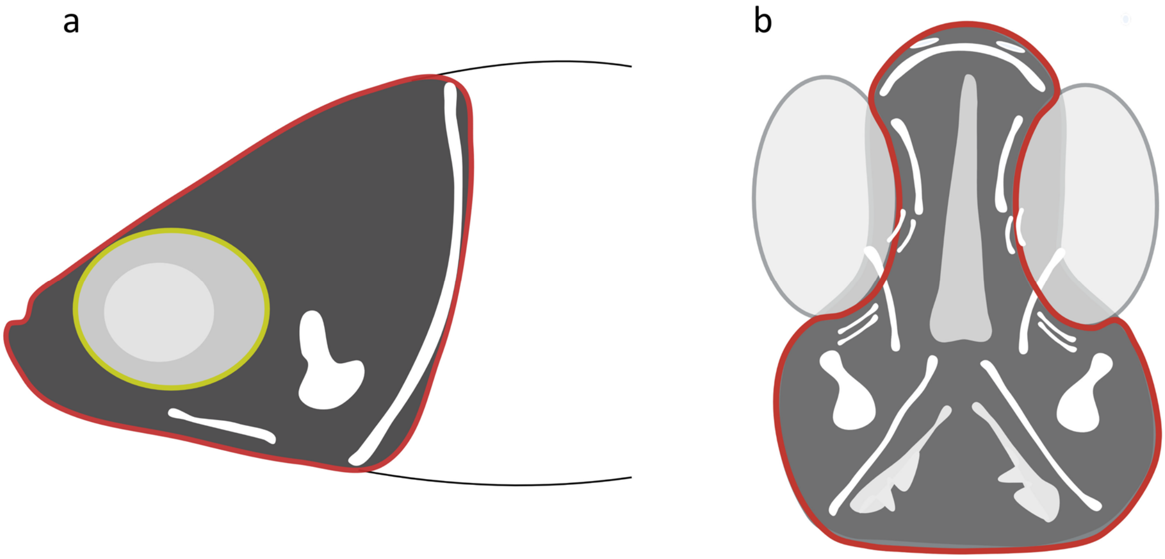

Supplement: Supplementary file 1 [file ijms-23-04748-s001.zip › ijms-1675302-supplementary.pdf]
